# Capsular Healing in Interportal and Periportal Capsulotomy Methods of Hip Arthroscopy

**DOI:** 10.1111/os.13132

**Published:** 2021-08-05

**Authors:** Zi‐yuan Li, Gang‐feng Hu, Zhi‐gao Jin, Qian Li, Zhuo‐yan Ling, Gao‐long Shi, Qi‐rong Dong, Zong‐gang Xie

**Affiliations:** ^1^ Department of Orthopaedics The Second Affiliated Hospital of Soochow University Suzhou China

**Keywords:** Capsular healing, Hip arthroscopy, Interportal capsulotomy, Outcomes, Periportal capsulotomy

## Abstract

**Objective:**

To evaluate the midterm outcomes and the capsular healing in patients who had interportal capsulotomy versus periportal capsulotomy of hip arthroscopy.

**Methods:**

Retrospectively reviewed 33 patients with labral tear received hip arthroscopy, with an average age of 41 (27‐67) years, including 13 cases of Cam deformity and three cases of Pincer deformity. All patients had positive sign of flexion adduction internal rotation or flexion abduction external rotation. With MRI and radiographic (CT, X plain) imageological examination. MRI showed that all patients had labral tear. Radiographic finding (CT, X plain) showed the pathological changes of acetabular and femoral neck osteophyte. One group with 23 patients were treated with periportal capsulotomy. Another group with 10 patients were treated with interportal capsulotomy. All patients did not close the capsule. Clinical outcomes were measured with the Hip Outcome Score Activities of Daily Living (HOS‐ADL) and the modified Harris Hip Score (mHHS), patient satisfaction measured with visual analogue scale (VAS). The healing of the capsule was evaluated by MRI. MRI showed continuous capsular indicated healing, discontinuous capsular indicated unhealing. Postoperatively 6 months, mHHS and HOS‐ADL were obtained. Randomized controlled trials were used in this study for analysis.

**Results:**

All patients were followed up with average time of 9.3 months(3‐29 months). The postoperative symptoms were obviously relieved, the VAS decreased from (4.9 ± 0.6) to (1.2 ± 0.2) after 3 months postoperative. Follow up 6 months post‐operation, patients in the interportal group, the mHHS and HOS‐ADL scores improvement were respectively 69.4 ± 9.3 & 70 ± 8.8 pre‐operation, and 92.5 ± 5.0 & 86.6 ± 5.4 post‐operation (*P* < 0.05); Patients in the periportal group, the mHHS and HOS‐ADL scores improvement were respectively 69.9 ± 15.8, 68.1 ± 15.0 pre‐operation, and 90.1 ± 9.3 & 86.7 ± 7.9 post‐operation (*P* < 0.05).The differences were statistically significant. Six months after operation, MRI showed that 23 patients with periportal capsulotomy, the capsule have healed, without other complications. Three of the ten patients with interportal capsulotomy were healed and seven were not.

**Conclusion:**

Interportal and periportal capsulotomy had good outcomes. The technique of periportal capsulotomy had little damage to the joint capsule. Although the capsule did not close, the capsule healed well in postoperative follow‐up. The nonunion rate of the joint capsule was high in the interportal capsulotomy without close the capsule.

## Graphical abstract

We chose periportal capsulotomy to cut the joint capsule. Establish anterolateral portal and Mid‐anterior portal, and the capsule were not closed after operation; Six months after operation MRI showed that the joint capsule healed well.

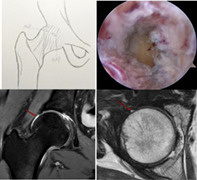

## Introduction

In recent years, with the development of the minimally invasive concept, hip arthroscopy has a significant growth^1^. Hip arthroscopy results in faster postoperative recovery, fewer complications compared to open hip procedures^2^. Joint instability after hip arthroscopy has attracted much attention as a serious complication^3^. During the operation of hip arthroscopy, it is necessary to destroy the joint capsule in order to establish operation path. Anatomy and biomechanics of hip joint capsule have been well realized. The joint capsule consists of three ligaments (iliofemoral ligament, pubofemoral and ischiofemoral ligament) and zona orbicularis, each of which has a different function^4^. The iliofemoral ligament (Y ligament of Bigelow) is the strongest joint capsule ligament, which can resist the anterior translation and external rotation of the hip joint. In the process of extension and external rotation, it also tightens in “screw home” mechanism^5^. The pubofemoral ligament provides restraint on external rotation, hyperextension and hyperabduction^6^. The ischiofemoral ligament limits the internal rotation because of its posterior position, as well as limits the adduction when the hip is flexed^7^. All these ligaments are static stabilizers of the hip joint. The zona orbicularis surrounds the entire femoral neck and acts as a locking ring around the femoral neck. This is the key structure of hip joint stability during distraction^8^. The joint capsule is an important anatomical structure to stabilize the hip joint, so the destruction of the joint capsule becomes an important factor for the instability of the hip joint^9^.

The most common techniques of capsulotomy are interportal and T‐shaped capsulotomies^10^. In the former method, a transverse incision in the capsule is made parallel to the labrum. The capsule is cut through between the anterolateral portal and the mid‐anterior portal. The interportal incision can be quite variable for different surgeons in terms of incision length and distance from the labrum. In the latter, a longitudinal incision along the femoral neck length is made in order to expose the femoral lesion. It is always accompanied with the interportal capsulotomy^11^. In general, interportal capsulotomy has a wider vision field of arthroscopy which can find and treat the central compartment and periphery compartment pathology, and resect the cam lesions near femoral head^12^. But it is difficult to manage the bone lesion in distal of femoral neck. Therefore, the T‐shaped capsulotomy was used to treat the lesion. T‐shaped technique has greater capsulotomy, wider view and more flexible usage of the instrument. In the condition, the surgeon can treat giant or more distal cam lesions and completely access the peripheral compartment^13^, especially the global type. These capsulotomies have a similar disadvantage, more defects of the capsule. Some surgeons try to adopt different methods to protect the capsule. They perform this by limited violation of the capsule through periportal capsulotomy without making a transverse incision between the portals^14^, ^15^. This way leads to little damage to the joint capsule, it' is easy to handle the repair of labrum injury and correct the Pincer type deformity. Sometimes, it is difficult to deal with Cam deformity, because the complete iliofemoral ligament and the orbicularis oculi band limit the exposure of the femoral neck. But most lesions can be treated by exchanging the observation portal and instrument portal. Because of the small defect of capsule, stability of hip joint is not be destroyed.

The destroyed capsule of interportal capsulotomy is about 3–5 cm, which is commonly extended in a “T” shape distally. This is carried out to provide widely visualization and full movement of instruments. Capsular closure is a controversial topic for hip arthroscopy surgeons, and the management of femoral acetabular impingement and labral tear has been studied more extensively. In the past, capsules were routinely released without closure. As reports of hip biomechanics and instability have become more common in the literature, hip arthroscopists performed numerous capsular closures, guided by patient characteristics^16^, the choices of capsular management include non‐repair capsulotomy, partial closure (only repair the longitudinal part of T‐capsulotomy or partially close the interportal incision) and complete closure^17^.

In this study, we used two types of capsulotomy. One is the periportal capsulotomy, another is interportal capsulotomy. The purpose of this study was to: (i) evaluate the midterm outcomes of two groups; (ii) observe the outcomes of capsular healing and compare the healing rate of two groups; and (iii) assessment the outcomes of patients with unclosed capsule.

## Materials and Methods

### Subjects

From December 2016 to January 2020, 33 patients, with an average age of 41 (27–67) years, were treated with hip arthroscopy, including 14 males, 19 females, nine on the left and 24 on the right.

The inclusion criteria were as follows: (i) patients presented with hip or groin pain, had positive sign of flexion adduction internal rotation or flexion abduction external rotation, magnetic resonance imaging findings of labral tear, arthroscopic labral repair was performed in all patients; (ii) the trial group with 23 patients were treated with limited incision of periportal capsulotomy, without closing the capsule; (iii) the control group with 10 patients were treated with interportal capsulotomy, without closing the capsule; (iv) primary clinical outcomes were measured with the Hip Outcome Score Activities of Daily Living (HOS‐ADL) and the modified Harris Hip Score (mHHS), patient satisfaction measured with VAS. The healing of the capsule was evaluated by MRI; and (v) randomized controlled study is designed to analyze the difference of curative effects of two capsulotomy methods and the capsule healing. The exclusion criteria were as follows: (i) patients with dysplasia; and (ii) patients with joint relaxation.

All the 33 cases were treated by hip arthroscopy due to labral tear, including 13 cases of Cam deformity and three cases of Pincer deformity. All 33 patients were followed up after operation (6–29 months), and modified Harris Hip scores and HOS‐ADL scores for hip improvement were recorded before and 6 months after surgery.

### Research Indicator

#### Modified Harris Hip Scores

The Modified Harris Hip scores (mHHS) scoring system mainly includes four parts as pain, function, deformity and range of motion. The score standard had a maximum of 100 points (best possible outcome). A total score < 70 is considered a poor score, 70–80 fair, 80–90 is good and 90–100 excellent.

#### Hip Outcome Score Activity of Daily Living Score

The hip outcome score activity of daily living score (HOS‐ADL) scoring system mainly include 19 items focuses on a wide range of functions from small activities such as putting on socks, standing and sitting, to more demanding activities like squatting, twisting and pivoting on the affected leg. The score standard had a maximum of 95 points (best possible outcome). A total score < 65 is considered a poor score, 65–75 fair, 75–85 is good and 85–95 excellent.

### Capsulotomy Technique

**Fig. 1:**
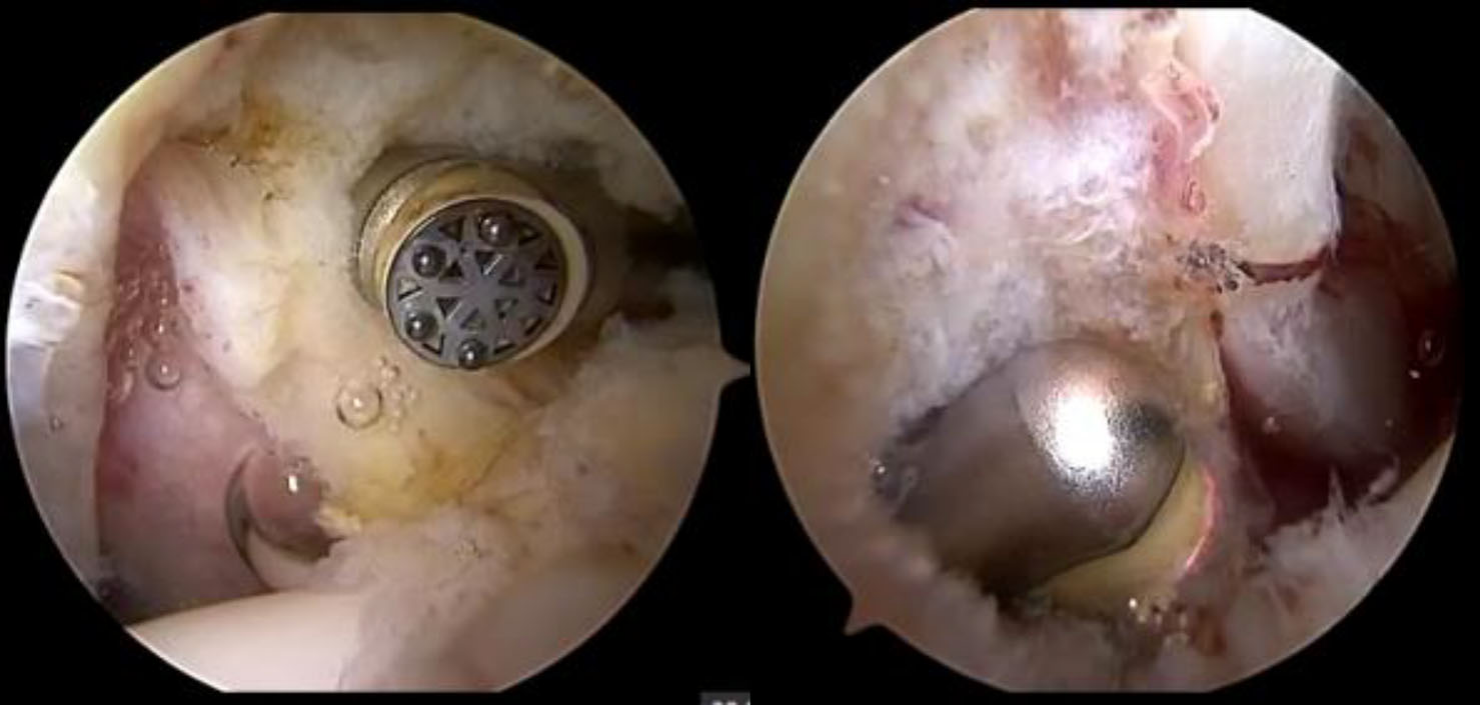
Limited violation of the joint capsule through the mid‐anterior and lateral portal.

**Fig. 2:**
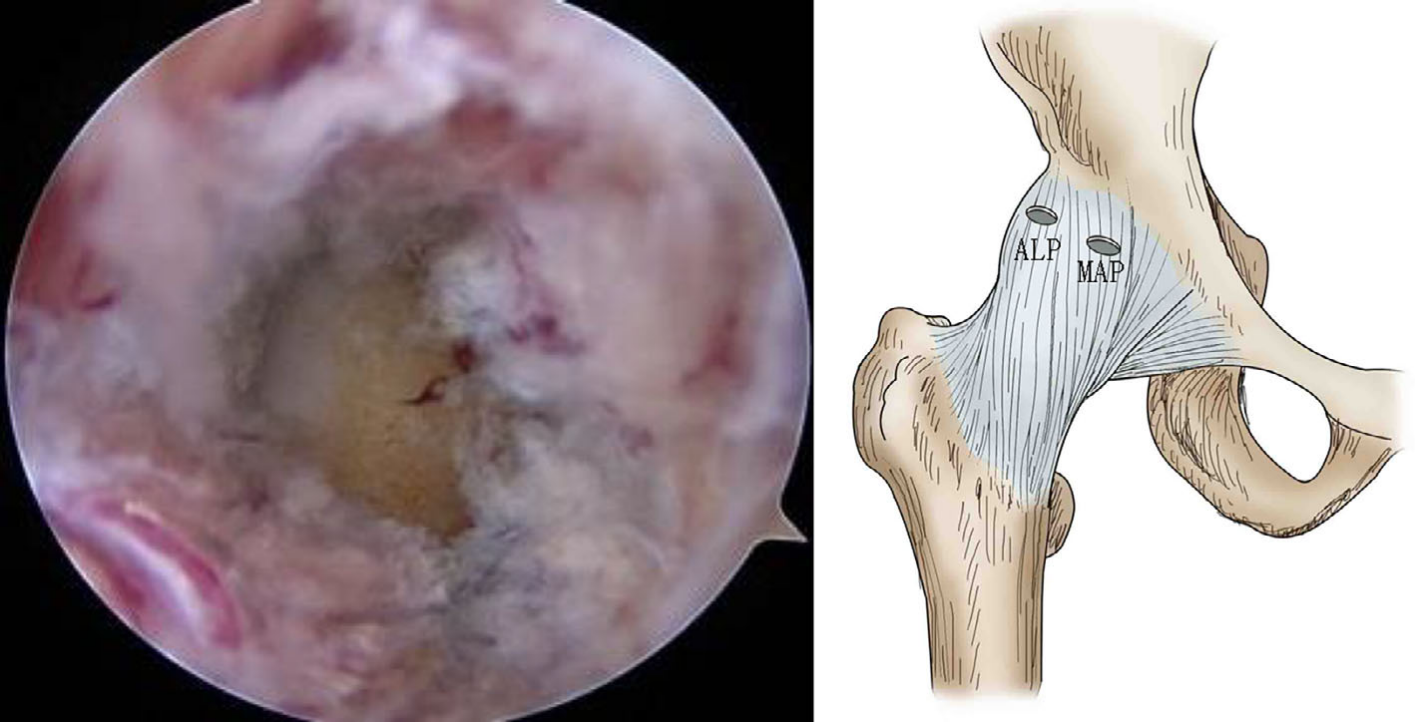
The finished portal incision. (ALP, anterolateral portal; MAP, mid‐anterior portal).

The periportal group patient lies supine on a traction table that allows dynamic leg positioning. Both feet are well secured and padded into the positioning boot, and a large padded perineal post is used to protect the perineum. The leg is in an internal rotation 20° position. The traction length of the hip joint is between 1–2 cm. The anterolateral portal is established under the guidance of the fluoroscope. A 70° arthroscope is inserted into the central compartment. Mid‐anterior portal is established under direct visualization with an arthroscope, which is about 1 cm distal to the labrum edge. A radiofrequency ablation device is inserted into the mid‐anterior portal, extending the portal by about 1 cm in size to carefully open through the full thickness of the capsule. The same procedure is repeated in the anterolateral portal, and no obvious restriction to the operating instruments without making a transverse incision between the two portals, preserving the iliac ligament. The incision capsule size of the portal was a1 cm (Fig. 1). The end of the operation showed a small incision and the capsule were not closed (Fig. 2). The interportal group patient use a similar method to establish anterolateral and mid‐anterior portal, the only difference is that the transverse incision was made between the two portals. The incision size of the joint capsule was 3–5 cm. The joint capsule was not closed at the end of the procedure.

### Hip Capsule Assessment

The oblique coronal plane demonstrated the most consistent defect and was clinically relevant in that it represented the iliofemoral portion of the capsule. Whenever a capsular gap was encountered, it indicated that the capsule had not unhealed. The presence of continuous capsular fibers indicated that the capsule has healed^18^.

All the patients received double crutch support for 4 weeks after the operation and prevented the excessive flexion and extension, abduction and abduction of the hip joint. The affected limb was partially loaded within 6 weeks and could be completely loaded after 6 weeks.

### Statistical Analysis

Changes in outcome scores from pre‐operative status to post‐operative were assessed using Wilcoxon signed‐rank test for nonparametric data (mHHS and HOS‐ADL). Outcome scores were summarized as means and standard deviations for quantitative variables. For comparisons between groups, the Mann–Whitney *U*‐test was used for nonparametric data. Statistical analyses were performed with SAS. Statistical significance for all comparisons was set at *P* < 0.05.

## Results

All patients were followed up with average time of 9.3 months (3‐29 months). The postoperative symptoms of 33 patients were significantly relieved, and the pain score (VAS) decreased from 4.9 ± 0.6 to 1.2 ± 0.2 after 3 months post‐operation.

### mHHS and HOS‐ADL

The modified Harris hip scores of hip joint improvement were 69.4 ± 9.3 pre‐operation and 92.5 ± 5.0 post‐operation (*P* < 0.05) in the interportal group; 69.9 ± 15.8 pre‐operation and 90.1 ± 9.3 post‐operation (*P* < 0.05) in the periportal group. The HOS‐ADL scores of hip joint improvement were 70 ± 8.8 pre‐operation and 86.6 ± 5.4 post‐operation (*P* < 0.05) in the interportal group; 68.1 ± 15.0 pre‐operation and 86.7 ± 7.9 post‐operation (*P* < 0.05) in the periportal group (Table 1).

**TABLE 1:** mHHS and HOS‐ADL score of two groups (mean±SD)

	mHHS			HOS‐ADL		
Groups	Preoperation	6‐months post‐operation	*P* value	Preoperation	6‐months post‐operation	*P* value
Interportal group	69.4 ± 9.3	92.5 ± 5.0	0.0002	70 ± 8.8	86.6 ± 5.4	0.0013
Periportal group	69.9 ± 15.8	90.1 ± 9.3	0.0001	68.1 ± 15.0	86.7 ± 7.9	0.0001

#### Results of Capsular Healing

**Fig. 3:**
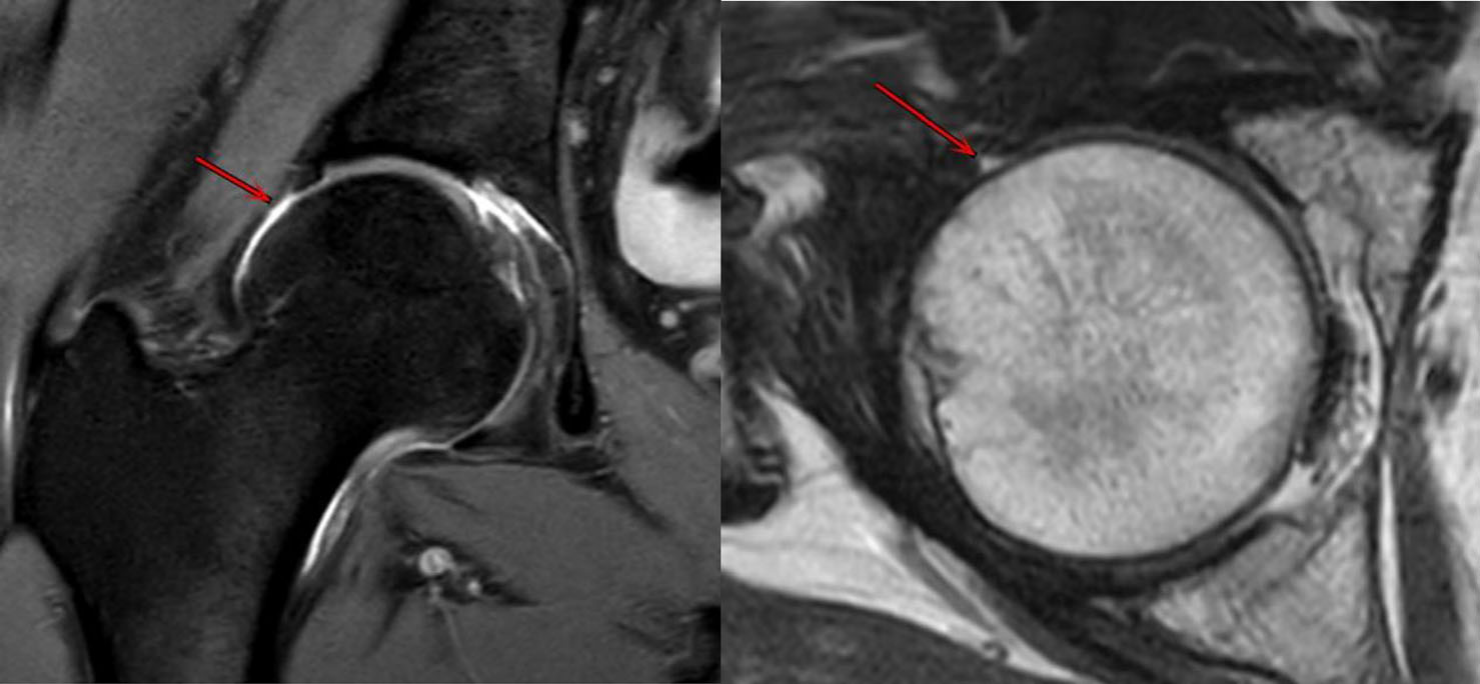
MRI shows 6 months after hip arthroscopy, the capsule has healed (red arrow).

**Fig. 4:**
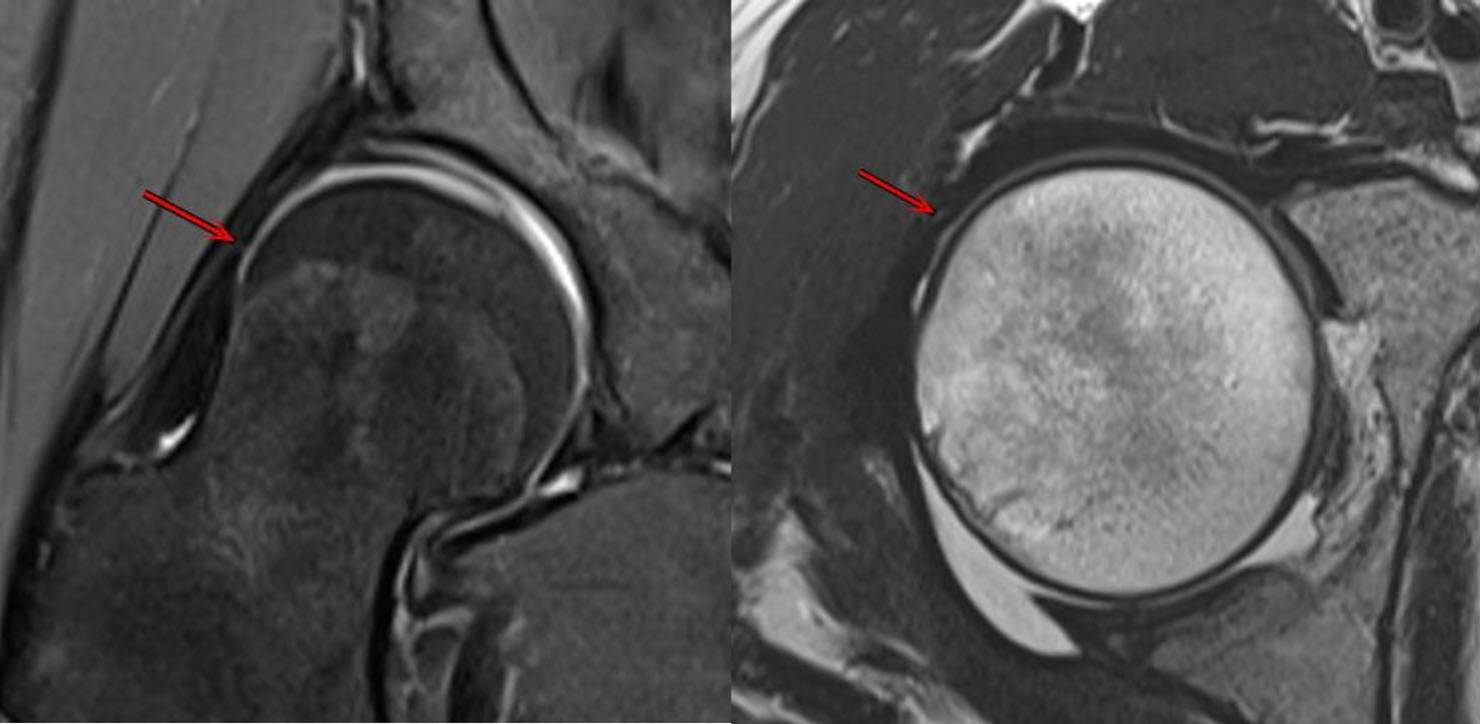
MRI shows 14 months after hip arthroscopy, the capsule has healed (red arrow).

**Fig. 5:**
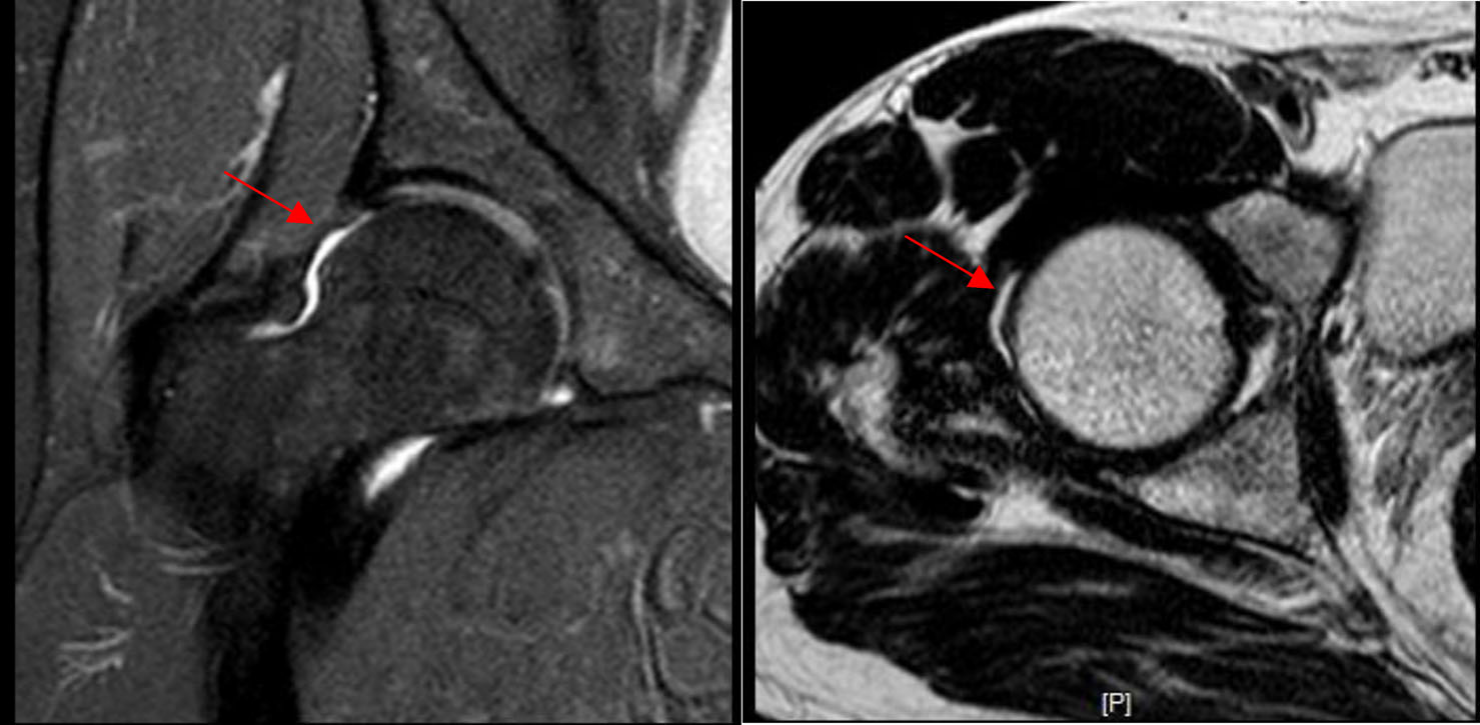
MRI shows 6 months after hip arthroscopy, the capsule has healed (red arrow).

**Fig. 6:**
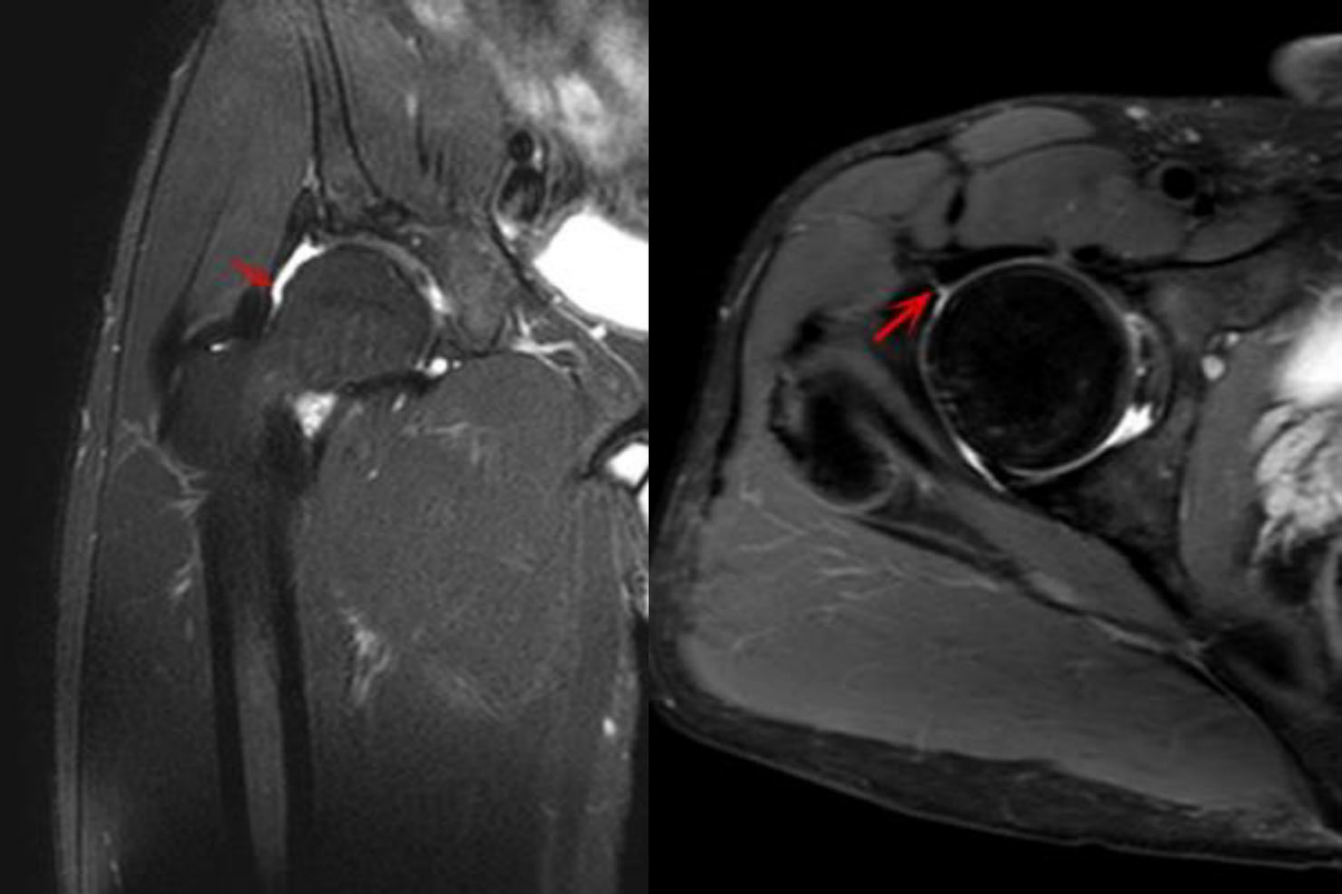
MRI shows 24 months after hip arthroscopy, the capsule has not healed (red arrow).

At 6 months post‐operative, MRI showed that in 23 patients with periportal capsulotomy, the capsule of all patients healed (Figs 3 and 4), the healed capsule can be seen at the arrow, which is thinner than the surrounding capsule and has obvious healing marks, with no other complications. Three of the 10 patients with interportal capsulotomy were healed (Fig. 5) and seven were not healed after 6 months post‐operation, the defective capsule can be seen at the arrow which is not filled with soft tissue, indicating that the capsule is not healed (Fig. 6).

## Discussion

The joint capsule is one of many structures that maintain the stability of the hip joint. It consists of the iliofemoral, pubofemoral, and ischiofemoral ligaments. The function of the joint capsule mainly prevents the dislocation and excessive rotation of the hip joint^4^, ^5^. Anterolateral portal and mid‐anterior portal are most commonly adopted for hip arthroscopy, with most surgeons using capsulotomy techniques, such as, interportal and T‐shaped capsulotomies, to provide freedom of visualization and full movement of instruments^19^. The large capsulotomy tends to bring about joint instability^20^, so many surgeons support the repair of capsular to maintain the stability of the joint^21^, ^22^. A variety of suture methods of joint capsule have been developed. However, the repair of joint capsule is technically demanding and may add substantial time to the surgical procedure and require appropriate suture tools. For patients without osseous structural abnormalities, some scholars have tried not to repair the joint capsule, resulting in different methods of capsulotomy^22^, ^23^.

Bolia *et al*. performing FAI correction and hip labral repair using an arthroscopic capsular incision of 2.5 cm, found that patients who undergo arthroscopic FAI correction and hip labral repair with repair of the capsule had higher HOS‐ADL and mHHS scores at midterm follow‐up compared to patients with capsular non‐repair. In addition, a lower rate of conversion to THA was seen in the repair group^24^. Harris *et al*. performed hip arthroscopy by opening the capsule with a T‐shaped incision and repaired the capsule completely after labral repair and other procedures^22^. Kraeutler *et al*. performed FAI correction using an arthroscopic small capsular incision (<3 cm) and randomly divided the patients into the repair group and the non‐repair group, MRI was performed again 6 and 24 weeks after surgery. Compared with the non‐repair group, the repair group found no significant increase in the percentage of consecutive hip capsules seen on MRI at 6 weeks after surgery. As assessed by MRI 24 weeks after surgery, all capsulotomy sites showed continuous healing of the joint capsule on MRI. Therefore, the joint capsule healed well after the operation through small incision of joint capsule^18^, ^25^. Domb *et al*. followed the joint capsule repair group and the unrepaired group for 2 years, found that the postoperative hip score was significantly improved in both groups, and there was no significant difference between the two groups. Compared with the unrepaired group, the joint capsule repair did not show better clinical efficacy. Because a small incision was chosen for capsulotomy, these results are not applicable to patients who receive a large capsulotomy (>3 cm) or “T” type capsulotomy, or to patients diagnosed with hip dysplasia^10^.

### Advantage and Disadvantage of Interportal Capsulotomy

The interportal capsulotomy technique is often used in hip arthroscopy. The advantage is that this surgical method has a wider surgical field of vision and is more convenient to deal with deformity, the disadvantage is that the damage to the joint capsule is great, especially to the iliofemoral ligament^26^, it is often necessary to suture the capsule^27^, ^28^. There are some scholars who believed that not suturing capsule has no effect on joint stability^10^, ^25^.

### Advantage and Disadvantage of Periportal Capsulotomy

The periportal capsulotomy technique we adopted had little damage to the joint capsule. The advantage is less damage of the hip joint. No incision was made between the anterolateral and mid‐anterior portal, and the incision of the joint capsule was about 1 cm. This technique preserves the integrity of the iliofemoral ligament or only minimally cuts the iliofemoral ligament, providing sufficient exposure to the hip joint, but only little damage to the stability of the hip joint, and does not require the closure of the capsule because of the small incision. The small incision reduces violation of the capsule, the adjacent innervation and musculature and relieve postoperative pain. The operation time is shorter, because the periportal capsulotomy does not need to close the capsule. Less fluid exudes to the surrounding muscle tissue^15^. The management of the acetabular side was not difficult, and the repair of labrum tear and the treatment of Pincer type deformity could be accomplished easily^29^. Sometimes the two portals may also be cut through due to the difficulty of operation, which requires the surgeon to be careful in the operation, as much as possible to reduce the joint capsule incision. Of course, there are disadvantages with the technique as it is difficult to deal with Cam deformity. Because the complete iliofemoral ligament and the orbicularis oculi band limit the exposure of the femoral neck and the range of movement of the operating instrument, the observation channel and the position of the operating instrument need to be changed repeatedly to complete the operation. For Cam deformities with a wide range, this technique is difficult to completely remove osteophytes, especially those in the distal part of the femoral neck, which often require a T‐shaped incision or cut‐through of two portals.

### Healing of Unclosed Capsule

MRI results of this study were obtained at more than 6 months after surgery. In the periportal capsulotomy group, MRI showed that the joint capsule incision had continuous fibrous tissue, indicating that the joint capsule had healed. It was suggested that although the joint capsule was not closed, the postoperative joint capsule healed well. In this group, all patients' capsular had healed. In the interportal capsulotomy group, MRI showed that seven of ten patients had unhealing capsule after 6 months operation. It indicated that the rate of non‐healing of the joint capsule was high without closure of the operation. Therefore, the joint capsule should be closed intraoperatively. The two methods of joint capsulotomy achieved good clinical efficacy, there is no obvious clinical difference between the two methods, but there is a great difference in integrity of anatomical structure, and there is a significant difference in the proportion of joint capsule healing. Whether there has difference of stability between the two groups need to be confirmed by more cases and long‐term follow‐up.

### Limitations

The deficiency of this paper is that the number of cases is limited and the time of postoperative MRI review is relatively scattered. The cases of the capsule healed well, but lack of MRI data at the same point of follow up, mostly only once, and relatively complete long‐term MRI data were not available. The cases of unhealed capsule, lacked long‐term observation and more cases are needed to analysis the stability of the hip joint.

### Conclusions

The hip arthroscopy with limited incision of periportal capsulotomy has satisfactory curative effect and less invasively trauma. It does not need suturing of the joint capsule and saves operation time, especially in the absence of appropriate capsular suture tools, which reduces the difficulty of the operation.
